# Atherosclerosis and Rheumatoid Arthritis: More Than a Simple Association

**DOI:** 10.1155/2012/147354

**Published:** 2012-09-13

**Authors:** Lorenzo Cavagna, Nicola Boffini, Giovanni Cagnotto, Flora Inverardi, Vittorio Grosso, Roberto Caporali

**Affiliations:** Department of Rheumatology, University and IRCCS Foundation Policlinico San Matteo, Viale Golgi 19, 27100 Pavia, Italy

## Abstract

In the last decades a large amount of evidence linked rheumatoid arthritis (RA) to atherosclerosis. In fact, RA patients have an increased risk of cardiovascular events that is not fully explained by other classic cardiovascular risk factors. RA and atherosclerosis may share several common pathomechanisms and inflammation undoubtedly plays a primary role. The proinflammatory cytokines such as tumor necrosis factor alpha and interleukin-6, involved in the pathogenesis of RA, are also independently predictive of subsequent cardiovascular disease (CVD). In RA, inflammation alters HDL constituents and the concentration of LDL and HDL, thus facilitating atherosclerosis and CVD events. On the other hand, also the increase of oxidative processes, frequently observed in RA, induces atherosclerosis. Interestingly, some genetic polymorphisms associated with RA occurrence enhance atherosclerosis, however, other polymorphisms associated with RA susceptibility do not increase CVD risk. Several other mechanisms may influence atherosclerotic processes in RA. Moreover, atherosclerosis may be directly mediated also by underlying autoimmune processes, and indirectly by the occurrence of metabolic syndrome and impaired physical activity. Finally, the effects of RA therapies on cardiovascular system in general and on atherosclerosis in particular are really wide and different. However, the starting point of every RA treatment is that disease control, or better remission, is the best way we have for the reduction of CVD occurrence.

## 1. Introduction

Rheumatoid arthritis (RA) and atherosclerosis are two inflammatory diseases strictly linked; in fact, although joint involvement is the prototypical feature of RA, atherosclerotic cardiovascular diseases (CVDs) are the major cause of mortality and morbidity in these patients [1, 2]. Therefore, the increased CVD risk occurs even early during the course of RA, being so intended as a possible preclinical manifestation of the disease [3]. On this basis, the understanding of commonly shared pathomechanisms is mandatory for the right treatment of RA, in order to reduce atherosclerosis and the subsequent impact of CVD, on these patients. Moreover, it's also important to evaluate the different effects of RA therapies (i.e., corticosteroids, NSAIDs, DMARDs, anti-TNF agents, and other biological drugs) on cardiovascular risk. All these aspects will be analyzed in this paper.

## 2. The Link between Atherosclerosis and Rheumatoid Arthritis

In the last years, a large amount of data improved the understanding of pathomechanisms leading to atherosclerosis appearance, thus allowing its classification among inflammatory disorders [4], similarly to RA. But this is not the only point that links atherosclerosis and RA; undoubtedly, smoke is the most evident one, being clearly involved in the appearance of both diseases [5, 6]. Moreover, it is well established that RA patients had an increased risk of cardiovascular events that is not fully explained by smoke and other classic CVD risk factors [3]. Therefore, atherosclerotic processes are increased in RA [7, 8], and subsequently also CVD risk. Inflammation plays a primary role in this relationship; in fact, in patients with recent onset polyarthritis, baseline CRP levels were independent predictors of CVD-related death, with an hazard ratio of 3.3, and this after adjusting for age, sex, smoking status, rheumatoid factor positivity, swollen joint counts, and Health Assessment Questionnaire score [9]. Furthermore, it is interesting to observe that the risk of CVD events, myocardial infarction in particular, is increased also in the 2 years preceding formal diagnosis of RA [10]; so, on this basis, it is possible to speculate that systemic inflammation may increase atherosclerosis before it affects the joints [11]. On the other hand, the longer the duration of disease, the higher the risk of plaques in the carotid artery [12] and CVD events [13], thus indicating that chronic RA-related inflammation increases the CVD risk and suggesting the need of early therapeutic intervention in these patients. Therefore, between the proinflammatory cytokines involved in the pathogenesis of RA, tumor necrosis factor (TNF) alpha and interleukin (IL)-6 are independently predictive of subsequent CVD events in these patients. In fact, these cytokines are released into the systemic circulation, with a large amount of systemic effects, in particular on endothelium [11]. The result is a cascade of alterations throughout our organism that leads to the proatherogenic profile that is prototypical of RA. 

## 3. Pathomechanisms Involved in Rheumatoid Atherosclerosis Appearance

RA-related inflammation may lead to atherosclerosis occurrence in several ways. The enhancement of oxidative modification of LDL, that has been linked to TNF-*α* action through the stimulation of superoxide secretion from monocytes and endothelial cells, is among involved processes [11]; moreover, also HDL constituents may be altered by the inflammation, thus losing their ability to remove cholesterol from atherosclerotic lesions and reducing their antioxidant activity [14]. On the other hand, not only the function but also the concentration of LDL and HDL is altered in RA; in particular small-dense LDLs are increased, whereas small-dense HDLs are decreased [11], thus leading to an unbalance toward atherosclerosis appearance. Therefore, HDL impairment may be related to paraoxonase (PON) activity reduction, that has been found in RA, in particular in patients with both active [15] and quiescent disease [16]; in fact, PON is a peculiar enzyme linked to HDL that binds and destroys oxidized lipids, thus reducing atherosclerosis occurrence [11]. Another aspect is the increase of oxidative processes, that in RA is demonstrated in several ways, for example, by the depletion of vitamins A and E and by the reduced degradation of asymmetric dimethyl-L-arginine (ADMA), an endogenous inhibitor of nitric oxide synthase (NOS) [11, 17]. Recently, the attention has been pointed out on Interleukin-17; this cytokine, involved in RA pathogenesis [18], may accelerate myocardial fibrosis and promote atherosclerosis in non-RA animal models [19]. Therefore, elevated circulating IL-17 levels have been detected in patients with acute coronary syndromes [20]. In RA patients, this cytokine influences microvascular function and arterial compliance, thus playing a significant role in development of endothelial dysfunction and CVD in the setting [21].

But although these factors are important, the “*sine qua non*” condition for atherosclerosis appearance is the occurrence of endothelial dysfunction, a feature frequently described in RA patients [22]; this generic term indicates the endothelial phenotypic alterations that appear in response to a large amount of noxious stimuli. The increased expression of adhesion molecules such as ICAM-1, VCAM-1 and E-selectin, the enhancement of pro-inflammatory cytokines (TNF-*α*, IL-1, IL-6, IFN-*γ*), and the upregulation of oxidative stress processes are the starting point of this condition [23]; moreover, also the increase of leptin and resistin (proatherogenic hormones) and the decrease of adiponectin (antiatherogenic hormones) may alter endothelial homeostasis in RA patients [22]. These features lead to an increase in endothelial permeability to lipoproteins and plasma constituents, with subsequent infiltration of lipids into the arterial wall and migration of monocytes and T-lymphocytes into the vessel intima [24]. Foam cells and fatty streaks appearance within the vessel wall is the consequence of these processes [25]. The inflammatory state leads to smooth muscle cells proliferation, that migrates into the lesion with subsequent vessel walls thickening and fibrotic tissue deposition. The result is the appearance of atherosclerotic plaques, that appear as a dynamic lesions, due to the large amount of ongoing modifying processes; but these lesions are also unstable, with an increased risk of rupture [24]. Therefore, recent data showed that endothelial progenitor cells (EPCs) action is altered in RA [26]; EPCs are mononuclear cells present in blood, bone marrow, and vessels that express specific endothelial markers and can help the repair of injured endothelium [11]. So, on this basis it is possible to speculate that in RA not only atherosclerosis formation mechanisms are increased, but also reparatory mechanisms are impaired. 

## 4. Genetic and Autoimmunity: Beside Classic Inflammation Pathways

After the identification through genome-wide association studies of a large amount of putative loci that may increase the risk of CVD in the general population, several researches have been addressed to the identification of a genetic backgrounds also for RA-related atherosclerosis. In particular, recent data evidenced that rs599839 A/G polymorphism (chromosome 1p13.3), previously associated with higher plasma total and LDL cholesterol levels and with an increased risk of CVD in general population, seems to increase the risk of endothelial dysfunction also in RA patients without evidence of overt CVD [27]. Another identified polymorphism, MIA3 rs17465637 A/C, enhances the risk of CVD also in RA, although only in case of concomitant dyslipidemia [28]; MIA3 protein is involved in leukocyte adhesive interactions with vascular endothelium, reduces attachment, and promotes migration of monocytes across the endothelium, thus leading to foam cells and fatty streak appearance with vessel walls [29]. Likewise to general population, acid phosphatase locus 1*C allele is associated with CVD events in RA population; as the authors state, this may result from the major production of the S isoform of low molecular weight phosphotyrosine phosphatase by this allele, which may influence the regulation of energy metabolism and the response to oxidative stress [30]. Therefore, a potential influence on CVD risk in RA patients has been suggested also for the CCR5Δ32 deletion [31], MTHFR 1298 A > C [32] and IL6-174 [33] gene polymorphisms.

Due to the relevance of TNF*α* in the inflammatory pathway of RA, several polymorphisms of this cytokine have been evaluated as a potential risk factors for atherosclerosis occurrence in this setting. In fact, both TNF*α* rs1800629 [34] and TNF*α* 1031 T/C [35] polymorphisms were associated with an enhancement of atherogenic processes in RA patients. Moreover, among the several HLA-DRB1 alleles involved in RA susceptibility, HLA-DRB1*0404 [36] is associated with an increased risk of endothelial dysfunction and CVD events in RA patients. In particular, patients with HLA-DRB1*0404 alleles had decreased endothelium-dependent vasodilatation with respect to other RA patients; interestingly, in these patients the authors do not find correlations between disease-related parameters (i.e., disease duration, activity parameters) and endothelial dysfunction. Moreover, the authors excluded also the occurrence of linkage disequilibrium with TNF microsatellite alleles, thus confirming that cardiovascular risk in RA may be partially genetically determined by HLA-DRB1*0404 alleles. However, up to now, the biological mechanisms underlying this association are not established, although antigen presentation and upregulated expression of HLA-DR*β*1 molecules on the endothelial cell wall may be involved [37]. 

It is interesting to observe that some polymorphisms may enhance CVD risk in RA patients carrying the HLA-DRB1*0404 allele, as recently demonstrated for endothelial nitric oxide synthase (NOS2A and NOS3) gene polymorphisms [38]. Another gene involved in RA susceptibility, the methionine sulfoxide reductase A (MSRA) gene, in particular the minor allele G, is associated with an increased risk of ischaemic heart disease in anticyclic citrullinated peptide antibodies (ACPAs) positive RA [39]. These data confirm that similarly to general healthy population, also in RA the genetic background influences atherosclerosis occurrence. However, some gene polymorphisms are associated with RA susceptibility but not with an increased CVD risk, as, for example, PTPN22, STAT4 and TRAF1/C5 [40], IL6R rs2228145 and IL6ST/gp130 rs2228044 [41], VEGFA rs2010963 and the rs1570360 [42], and MHCIITA rs3087456 and rs4774 [43]; on the other hand, potentially involved polymorphisms, such as those of macrophage migration inhibitory factor-173, do not link with RA susceptibility and atherosclerosis occurrence [44]. These results by itself are very intriguing, because they indicate that the pathway of atherosclerosis in RA is really complex, being influenced by several factors, that may transcend inflammation and genetic background. In fact, atherosclerosis in RA may be mediated also by underlying autoimmune processes; in particular, antibodies against oxidized low-density lipoprotein have been found to be associated with subclinical atherosclerosis in recent-onset RA [45–47]. Moreover, anti-apoA-1 IgG are increased in RA patients, being also significant predictors of CVD in these patients [48]. The APCAs, that are important serological markers of RA, have been assessed as a possible markers of atherosclerosis appearance in this setting. In particular, Gerli et al. [49] showed that RA patients with detectable circulating APCA had higher intima-media thickness (IMT) at internal carotid arterial wall than patients without evidence of these antibodies, thus linking ACPA positivity and subclinical atherosclerosis in RA. Recently, the attention has been pointed out on another citrullinated antibody, the antimodified citrullinated vimentin (anti-MCV); changes in serum levels of this antibody correlate with changes in atherogenic ratios (total cholesterol/HDL-C and LDL-C/HDL-C), apolipoprotein A-I, and carotid IMT, thus becoming a possible marker of subclinical atherosclerosis in RA [50]. But the autoantibodies may be sometimes protective, as demonstrated for antibodies against phosphorylcholine (anti-PC) [51], that probably play a role in the clearance of atherosclerotic plaques [52]. In fact, low IgM anti-PC levels are associated with an increased occurrence of carotid plaques in RA patients [53]. 

So, taken together, these data indicate that between RA and atherosclerosis there is a very close link, that is not limited to inflammation processes and involves also genetic and autoimmunity; in fact, this link is so deep that some authors include atherosclerosis among the extra-articular manifestations of RA [11]. 

## 5. Other Factors Involved in Atherosclerosis Appearance

In order to better understand the increased CVD risk in RA we should take into account also other indirect factors. In fact, metabolic syndrome is common in both early and long-standing RA, as indicated by the increase of waist circumference and blood pressure, by the occurrence of dyslipidaemia and abnormal visceral fat distribution [54, 55]. Therefore, also the impaired physical activity may affect the risk of CVD in these patients; in fact, low physical activity in RA women is associated with increased levels of oxidized low-density lipoprotein (oxLDL) and insulin, with reduced levels of HDL, Apo A1 and atheroprotective natural anti-PC, and, in particular, with insulin resistance [56]. This latter in particular is associated with impaired vascular insulin signaling and blunted vascular effects of insulin, that lead to atherogenesis appearance, although through mechanisms that are not completely established [57].

## 6. Atherosclerosis and RA Therapies

The effects of RA therapies on cardiovascular system in general and on atherosclerosis in particular are really wide and different. In fact, RA treatment comprehends drugs that may either increase or reduce CVD risk [58]. However, the starting point of every RA treatment is that disease control, or better remission, is the best way we have for the reduction of cardiovascular morbidity and mortality in these patients [59]; but RA patients always ask for pain control, so that we should take into account the drugs used for pain control.

### 6.1. Nonsteroidal Anti-Inflammatory Drugs (NSAIDs)

NSAIDs are largely used in RA as a painkillers; actually two main classes of NSAIDs are available: COXIBs, that act selectively on inducible COX-2, and classic NSAIDs, that block both COX-1 and COX-2. These classes of drugs have been recently linked to an increased CVD risk, first for rofecoxib [60], with subsequent reduced trend in COXIBs prescription [61], and then also for other COXIBs and NDSAIDs [62]. However, the relationship between COXIBs/NSAIDs and atherosclerosis occurrence is not clearly established; in particular, some authors hypothesized that COXIBs may promote the early appearance of atherosclerosis, whereas in case of more advanced stages, they may demonstrate protective/antiatherogenic properties [63]; it is interesting to observe that this dualism has been confirmed also in subsequent studies for both COXIBs and NSAIDs [64–67].

### 6.2. Corticosteroids

Corticosteroids (CTs) are powerful anti-inflammatory agents widely used in the treatment of RA [68]. Although long-term CTs use may be associated with a dose-related increased risk of CVD, due to effects on blood pressure, insulin resistance, lipid profile, body weight, and fat distribution [69–71], up to now there is no evidence that low-dose CTs may influence atherosclerosis appearance in RA [72]. Moreover, other data showed that anti-inflammatory and antiproliferative actions on vessel walls of low-term CTS therapy may reduce first atherosclerosis occurrence and then CVD risk [58]. 

### 6.3. Disease Modifying Antirheumatic Drugs (DMARDs)

The term DMARDs indicates a wide group of drugs potentially able to inhibit the occurrence/progression of articular damage in RA patients [73]. Therefore, DMARDs may reduce also CVD risk by influencing atherosclerotic processes directly through inflammation; but in order to obtain this goal, the early identification and treatment of patients with RA are crucial [74]. Among DMARDs are listed drugs such as methotrexate (MTX), leflunomide (LFN), sulphasalazine (SSZ), cyclosporine (CsA), and hydroxychloroquine (HCQ). 

MTX is today the anchor DMARDs for RA treatment; moreover, recent studies showed that although increasing serum homocysteine levels [75], MTX reduces CVD-related mortality and morbidity in RA with respect to other DMARDs [58, 76, 77]; this suggests that reducing RA inflammation, MTX may also reduce collateral damage such as atherosclerosis. Therefore, MTX-related atherogenesis reduction has been confirmed also in a recent experimental model [78]. Data on other DMARDs are scanty; LFN may improve vascular function through the inhibition of NF*κ*B signal transduction pathway in endothelial cells, the reduction of subendothelial migration of peripheral blood mononuclear cells, and, finally, to the impairment of antigen presenting dendritic cells [58]. However, despite these potential beneficial effects on atherogenesis, LFN-related arterial hypertension may increase CVD risk [79]. On the other hand, despite the established anti-inflammatory effects, also CsA has been associated with an increased susceptibility to atherosclerosis and development of hyperlipidemia; in fact, CsA demonstrates complex effects on lipoprotein metabolism and bile acid production and affects endothelial cells, smooth muscle cells, and macrophages, all critical for atherosclerotic process occurrence [80]. The effects of SLZ on atherosclerosis have been recently evaluated, although in patients with coronary artery disease without RA; the final findings of the study suggested that SLZ is not the optimal anti-inflammatory treatment for reversing endothelial dysfunction in cardiovascular disease [81]. Finally, hydroxychloroquine exerts an antithrombotic effect and improves glucose and lipid profiles in treated patients, being a potentially protective factor against atherosclerosis appearance [58].

### 6.4. Biological Agents

#### 6.4.1. Anti-TNF Agents (Infliximab, Etanercept, Adalimumab, Golimumab, and Certolizumab)

These drugs act through the inhibition of TNF alpha, a proinflammatory cytokine playing a primary role in RA appearance [82]; however, as previously described, TNF alpha has been implicated also in the pathogenesis of RA-related atherosclerosis. According to these suggestions, in the last decade numerous publications suggested that TNF blockers exert significant effects on the vasculature [83, 84] and decrease the incidence of CVD in RA patients [85]. The cardioprotective effect of TNF inhibition in RA may be related to several factors, as, for example, the increase of HDL levels; therefore, these drugs do not affect LDL levels or atherosclerotic index (i.e., TC/HDL ratio) [86]. On the other hand, these drugs may reduce significantly insulin levels and the insulin/glucose index, as well as improve insulin resistance [58, 87] and also a dramatic reduction of resistin, an adipokine that showed strong correlation with C-reactive protein, was observed following infliximab infusion in RA patients undergoing this therapy because of severe disease [88]. Likewise, improvement of endothelial function following anti-TNF-alpha administration has been observed in RA patients with severe disease refractory to conventional DMARDs therapy [89, 90].

 It is also important to remember that levels of circulating adhesion molecules, such as serum E-selectin and intercellular adhesion molecule-1, are decreased following these treatments [58]. However, despite this behavior, the use of TNF-blockers in patients with severe chronic heart failure may have detrimental effects on cardiac function, and this despite the increase of circulating levels of TNF observed; consequently, severe heart failure contraindicates anti-TNF treatment in patients with RA [91].

#### 6.4.2. Other Biological Drugs (Tocilizumab, Abatacept, and Rituximab)

Today a large number of non-anti-TNF biological drugs are available for RA treatment. The targets of these drugs are very wide; tocilizumab (TCZ) acts through the inhibition of IL-6, another proinflammatory cytokine that may contribute to atherosclerosis processes; in fact, TCZ improves endothelial function and aortic stiffness in RA and this despite the increase of total and LDL-cholesterol [58]. Abatacept is a fully human soluble fusion protein consisting of the extracellular domain of human CTLA-4 and the modified Fc portion of human IgG1; to date, there is no data on the effects of this drug on atherosclerosis. Rituximab (RTX) is a chimeric monoclonal antibody against CD20 depleting B cells in peripheral blood; also RTX data are scanty, although, at least in short term, this drug seems to improve endothelial dysfunction, carotid atherosclerosis, and lipid profile in RA [92, 93].

## 7. Conclusions

Rheumatoid arthritis and atherosclerosis are strictly linked in several ways; this link is so strong that atherosclerosis may be considered an “extra-articular manifestation” of the disease, leading to an increased risk of CVD [11]. Moreover, the impact of this “extra-articular manifestation” on patients survival is of primary importance, being in fact CVD, the main prognostic factor in this setting [1]. So it is important to screen and monitor RA patients for the occurrence of existing traditional-risk factors for CVD appearance, in order to reduce the impact on cardiovascular system, as suggested in the recently published EULAR evidence-based recommendations for cardiovascular risk management in patients with RA [94]. Therefore, according to literature evidence, it will be of primary importance that all risk stratifications models used to calculate CVD risk in general populations consider also RA among risk factors [95]. Additional tools such as the use of carotid ultrasonography in patients with RA that exhibit an intermediate risk have recently been suggested [96]. However, further studies are needed to better establish the cardiovascular risk of patients with RA. Regarding the treatment of atherosclerosis in RA patients, beside the classical approach [97], RA control, or better remission, should be considered the reference therapeutic strategy for the reduction of CVD risk in this setting [94].
